# Silencing immune-infiltrating biomarker CCDC80 inhibits malignant characterization and tumor formation in gastric cancer

**DOI:** 10.1186/s12885-024-12451-y

**Published:** 2024-06-13

**Authors:** MeiHong Yu, Jingxuan Peng, Yanxu Lu, Sha Li, Ke Ding

**Affiliations:** 1 Department of Gastroenterology, The Second Xiangya Hospital of Central South University, Changsha, China; 2 Research Center of Digestive Disease, Central South University, Changsha, China; 3 Department of Urology, First Affiliated Hospital of Jishou University, Jishou, Hunan China; 4 Xiangya Stomatological Hospital & School of Stomatology, Central South University, Changsha, China; 5 Department of Burns and Reconstructive Surgery, Xiangya Hospital of Central South University, Changsha, China; 6 Department of General Surgery Thyroid Specialty, The Second Xiangya Hospital of Central South University, Changsha, China

**Keywords:** Immune invasion, Gastric cancer, CCDC80, Prognosis, Stemness

## Abstract

**Objective:**

Tumor immune infiltration leads to poor prognosis of gastric cancer patients and seriously affects the life quality of gastric cancer patients. This study was based on bioinformatics to screen prognostic biomarkers in patients with high degree of immune invasion of gastric cancer. Meanwhile, the action of biomarker CCDC80 was explored in gastric cancer by cell and tumorigenesis experiments, to provide reference for the cure of gastric cancer patients.

**Methods:**

Data sets and clinical massage on gastric cancer were collected from TCGA database and GEO database. ConsensusClusterPlus was used to cluster gastric cancer patients based on the 28 immune cells infiltration in ssGSEA. R “Limma” package was applied to analyze differential mRNAs between Cluster 1 and Cluster 2. Differential expression genes were screened by single factor analysis. Stemness markers (SERPINF1, DCN, CCDC80, FBLN5, SPARCL1, CCL14, DPYSL3) were identified for differential expression genes. Prognostic value of CCDC80 was evaluated in gastric cancer. Differences in genomic mutation and tumor microenvironment immune infiltration were assessed between high or low CCDC80. Finally, gastric cancer cells (HGC-27 and MKN-45) were selected to evaluate the action of silencing CCDC80 on malignant characterization, macrophage polarization, and tumor formation.

**Results:**

Bioinformatics analysis showed that CCDC80, as a stemness marker, was significantly overexpressed in gastric cancer. CCDC80 was also related to the degree of gastric cancer immune invasion. CCDC80 was up-expressed in cells of gastric cancer. Silencing CCDC80 inhibited malignant characterization and subcutaneous tumor formation of gastric cancer cells. High expression of CCDC80 was positive correspondence with immune invasion. Silencing CCDC80 inhibited M2 polarization and promoted M1 polarization in tumor tissues. In addition, gastric cancer patients were likely to have mutations in CDH1, ACTRT1, GANAB, and CDH10 genes in the High-CCDC80 group.

**Conclusion:**

Silencing CCDC80, a prognostic biomarker in patients with immune invasion of gastric cancer, could effectively inhibit the malignant characterization, M2 polarization, and tumor formation of gastric cancer.

**Supplementary Information:**

The online version contains supplementary material available at 10.1186/s12885-024-12451-y.

## Introduction

Gastric cancer is the third of cancer-associated death worldwide, and usually has a poor prognosis [1, 2]. Due to the poor survival of advanced gastric cancer patients, it is imperative to study new cure methods to enhance the survival of gastric cancer [3]. The implementation of biomarker tests, particularly the analysis of HER2 status or PD-L1, had a significant impact on clinical practice and patient care [4]. Both cancer and the host immune system contain very diverse ecosystems to cause the functional analysis of immunogenomics [5]. With a better understanding of gastric cancer immunogenomics, further research and a better understanding of immune system function will promote immunotherapy in the future.

Asian Cancer Research Group (ACRG) and Genomic data from the Cancer Center Genome (TCGA) classifications are being applied to identify molecular subtypes to screen biomarker in cancer subpopulations [6]. In advanced disease, in addition to chemotherapy, only trastuzumab or immune checkpoint inhibitors (opdivo and pomerlizumab), have shown consistent efficacy in PD-L1-positive or HER2-positive tumors, respectively [7]. Targeted therapies based on biomarkers are rapidly developing, including MSI/dMMR, HER2, and PD-L1 [8, 9]. In addition, some drugs target the dryness of cancer by regulating genes, providing new strategies for the treatment of cancer [10]. Thus, screening for stemness biomarkers associated with gastric cancer immune infiltration could help develop new therapeutic strategies.

The genes associated with the stem characteristics of cancer can be used to analyze potential immune pathways and evaluate the prognosis of patients with colorectal adenocarcinoma [11]. Immune checkpoint inhibitors (ICIs) and anti-angiogenic agents have shown activity in advanced gastric cancer and initial efficacy in neoadjuvant/conversion settings [12]. Immunotherapy drugs, such as pembrolizumab, nivolumab and atezolizumab, were accelerated as the checkpoint inhibitor category by FDA [13]. Immunotherapy based on ICIs is considered a promising approach for the treatment of gastric adenocarcinoma, but therapeutic efficacy is limited by the complex tumor immune microenvironment of gastric adenocarcinoma [14]. Therefore, exploring cancer cell stemness markers associated with tumor microenvironment immunity may contribute to the development of new adjuvant chemotherapy methods for gastric cancer.

In humans, CCDC80 is expressed in various tumor cell lines and tissues, and is a potential oncogenic factor [15]. CCDC80 as a dryness marker is associated with immune score and immune infiltration in patients with OC [16]. CCDC80 is one of the predictive biomarkers for muscle-invasive bladder cancer patients sensitive to ICIs [17]. This paper aims to analyze prognostic biomarkers (CCDC80) related to immune infiltration in patients with gastric cancer by bioinformatics method. The role of marker CCDC80 was investigated by cell and tumorigenic experiments, which provided reference for the treatment of immunoinfiltration subtypes in gastric cancer patients.

## Methods

### Data source and immunoinfiltration subtype analysis

The Cancer Genome Atlas (TCGA) gastric cancer database (Supplementary Dataset File 1) and Gene Expression Omnibus data sets (GEO: GSE62254, Supplementary Dataset File 2) were applied to obtain the stomach of RNA sequencing (RNA - seq) data with complete clinical massage. Fragment (FPKM) values per kilobase of RNA sequencing data were obtained by transcript values per kilobase for subsequent analysis. In the light of 28 immune cells infiltration in ssGSEA, ConsensusClusterPlus R package for unsupervised clustering was applied to obtain gastric cancer subtypes (cluster1 and cluster2, cutoff = 4.20). Differential genes (abs(logFC) > 1 & *p* < 0.05) were identified by single factor analysis in TCGA dataset (*p* < 0.01). Stemness markers with SERPINF1 [18], DCN [19], CCDC80 [20], FBLN5 [21], SPARCL1 [22], CCL14 [23] and DPYSL3 [24], were identified for differential expression genes. Among them, CCDC80 is one of the differential genes.

### Immunoinfiltration analysis

Tumor immune estimation resource2.0, estimation of stromal and immune cells in malignant tumor tissues expression (ESTIMATE) algorithm, MCPcounter algorithm were applied to estimate the immune infiltration in gastric cancer [25, 26]. The enrichment score was calculated by using a single sample Genome Enrichment Analysis (ssGSEA) with R Genome Variation Analysis (GSVA) package.

### Function analysis and tumor mutation

Kyoto Encyclopedia of Genes and Genomes (KEGG) and Gene Ontology (GO) were applied for gene enrichment analysis through over representation analysis (ORA). Maftools R package (https://mp.weixin.qq.com/s/bKcQQ4pJorYDEmf150r2zg) was used to analyze tumor mutation [27].

### Cell experiment and grouping

HGC-27 cells (AW-CNH127, Abiowell) and HS746T cells (AW-CCH231, Abiowell) were incubated in DMEM + fetal bovine serum (10%, FBS) + penicillin streptomycin (1%, P/S) medium. GES-1 cells (AW-CNH199, Abiowell), MKN-45 cells (AW-CCH276, Abiowell) and MKN-74 cells (AW-CCH277, Abiowell) were cultured in 1640 + 10% FBS + 1% P/S medium. The expression of CCDC80 was confirmed using GES-1, HGC-27, HS746T, MKN-45, and MKN-74 cells. HGC-27 and MKN-74 cell lines were selected and randomly divided into Control, si-NC, and si-CCDC80 groups to investigate the function of CCDC80. Cells in the si-NC group, and si-CCDC80 group were transfected through Lipofectamine™2000 with si-NC, and si-CCDC80, respectively.

### Tumor formation in nude mice

Twelve BALB/c nude mice (female, 4 ~ 6 weeks old, SPF grade, average weight 18 g) were provided by Hunan SJA Laboratory Animal Co., Ltd. Mice were housed in a standardized environment with access to free food and water. Mice were set into Control, si-NC, and si-CCDC80 groups for subcutaneous tumor experiments, with 3 mice in each group. In brief, 2 × 10^6^ of HGC-27 cells were suspended in 100 µL PBS and injected subcutaneously in BALB/c nude mice [28]. The si-NC and si-CCDC80 were injected intratumorally in a volume of 100 µL once every 3 days. After 7 days, tumor length and width were measured every other day until the 28th day. The volume of the tumor was calculated by formula: V = length × width^2^ × 0.5. On the 28th day, each group of nude mice was placed in a carbon dioxide euthanasia chamber. CO_2_ was infused into the chamber at a rate of 20% of the chamber volume per minute (5.8 L/min, 5 min). When the animals were observed to be motionless with dilated pupils, the CO_2_ cylinder valve was closed. Then, the animals were observed for an additional 2–3 min to confirm death. The tumor tissue was photographed and collected for subsequent analysis.

### Cell counting Kit-8 (CCK8)

Cells from the above groups were digested and counted using trypsin (AWC0232, Abiowell). The cells were seeded in a 96-well plate with 5 × 10^3^ cells per well, 100 µL per well. After the cell’s attachment, 10 µL of CCK8 (AWC0114s, Abiowell) was added. Cells were then incubated at 37 °C, 5% CO_2_ for 4 h. Absorbance at 450 nm was measured using the microplate reader (MB-530, HEALES).

### Transwell

To detect cell invasion, serum-free DMEM medium (100 µL) diluted with Matrigel (200 µg/well, 354,262, BD) was added. Plate was incubated for 30 min. Supernatant was removed. 500 µL of 10% FBS medium was added to the lower chamber for cell invasion and migration detection. The treated cells were digested with trypsin to obtain single cells. 100 µL of cells (2 × 10^6^ cells/mL) were added for incubation. The upper chamber was removed and placed in a new well containing PBS for washing. The cells on the upper chamber were cleaned with a cotton ball. Membrane was fixed with paraformaldehyde (4%, AWI0070a, Abiowell) for 20 min. The membrane was stained with crystal violet (0.1%, AWC0333, Abiowell) for 5 min and washed with water five times. Membrane was placed on a glass slide, and observed under an inverted microscope (DSZ2000X, Cnmicro) to observe the outer surface cells of the upper chamber.

### RT-qPCR

Total cellular RNA was extracted through Trizol. The RNA concentration was measured using a UV spectrophotometer. The absorbance values were obtained at 260 nm ~ 280 nm. The cDNA was synthesized using the reverse transcription kit (CW2569, CWBIO, China). Amplification reactions were analyzed using UltraSYBR Mixture kit (CW2601, CWBIO, China) and a PCR instrument (PIKOREAL96, Thermo, USA). The amplification program consisted of 95 °C for 10 min, followed by 40 cycles of 95 °C for 10 s and 60 °C for 30 s. Target gene (Table 1) expression in each sample compared to the control group were calculated using the 2^−ΔΔCt^ method.

**Table 1:** Primer sequence

Gene	Sequence	Length
CCDC80	F: AGCCAGACTATGGGGGAAAG	154 bp
	R: GTTTCTCCTCGGTCTCGTGT	
JAK1	F: TTGCCAGAACTGCCCAAGGAC	160 bp
	R: CGCTGCTGTCACAAATGGTCT	
JAK3	F: CCTTCGAAAGTCCAGGGTCC	77 bp
	R: GATCAGGGGCGTCTCTTCAC	
STAT2	F: ATTCTGCCGGGACATTCAGG	244 bp
	R: GCAGAAGACATCCTGCTGGT	
STAT3	F: CTCTTACTTCTCCAGCAACACT	176 bp
	R: ATACATGCTACCTAAGGCCAT	
STAT4	F: ATCCTGGCTACCCATCCCTT	108 bp
	R: CACTGAGACATGCTGGAGCC	
STAT5A	F: AATGAACAGAGGCTGGTCCG	201 bp
	R: CCTCAGGCTCTCCTGGTACT	
STAT5B	F: GACCCAGCGCAGGCAA	160 bp
	R: CTTGGAGCTGCTGAGCTTGT	
β-actin	F: ACCCTGAAGTACCCCATCGAG	224 bp
	R: AGCACAGCCTGGATAGCAAC	

### Western blot

After the experiment, cell samples were collected and added RIPA (AWB0136, abiowell) lysis buffer to extract proteins. Protein concentration was determined by BCA method. Proteins samples were separated using SDS-PAGE. The separated proteins were transferred to a polyvinylidene difluoride membrane using trans-blot electrophoretic transfer (AWC0114, Abiowell) and dried at room temperature for at least 1 h. The membranes were cleaved (1–2 intervals above and below) based on the molecular weight of the target protein before hybridization with antibodies. Then, membrane was incubated with anti-CCDC80 (PA5-45821, 1 µg/mL, Thermo Fisher, USA) and reference antibody anti-β-actin (66009-1-Ig, 1:5000, Proteintech, USA). Membrane was incubated at 37 °C for 90 min with anti-IgG (AWS0001, 1:5000, Abiowell, China) and anti-IgG (AWS0002, 1:5000, Abiowell, China). Finally, membrane was visualized using superECL plus ultra-sensitive luminescence solution (AWB0005, Abiowell, China) and analyzed using ChemiScope6100 software (Clinx, China). The left and right edges of the blots as well as the marker were clearly visible. All blots with 3 replicates were shown in Supplement Fig. 1.

### Immunohistochemistry (IHC)

Tumor tissue was fixed in 10% neutral formalin. Tissues were sliced into 5 μm sections through a slicer (YD-315, Zhejiang Jinhua Yidi Testing Equipment). The sections were baked for 12 h at 60℃. Subsequently, the sections were treated with xylene and gradient ethanol. The sections were immersed in citrate buffer (0.01 M, pH 6.0) and heated in a microwave until boiling for 20 min, then cooled to room temperature. Sections were washed with PBS (0.01 M, pH 7.2–7.6) for 3 min, repeated 3 times. The sections were treated with 1% periodic acid. The sections were incubated with appropriately diluted anti-PCNA (10205-2-AP, 1:500, Proteintech, USA) overnight at 4℃. The sections were incubated with 50–100 µL of anti-IgG antibody-HRP polymer. Section was incubated with pre-made DAB working solution for 1–5 min. The sections were counterstained with hematoxylin and rinsed with PBS. Finally, the sections were observed by a microscope.

### Flow cytometry

Tumor tissues were ground to obtain cells. The cells were washed with PBS and then re-suspended in 100 µL of basal culture medium. The cells were stained with CD11b antibody (17-0112-82, eBioscience) in the dark for 30 min. To detect the proportion of M1 cells, the cells were stained with CD86 antibody (12-0862-82, eBioscience) in the dark for 30 min. To detect the proportion of M2 cells, the cells were sequentially fixed at 4 °C for 30 min with 1 mL of cell fixation solution and then permeabilized for 30 min with 1× permeabilization buffer. Subsequently, the cells were stained with CD206 antibody (12-2061-82, eBioscience) in the dark for 30 min. Finally, the cells were re-suspended in 200 µL of staining buffer and analyzed using a flow cytometer (A00-1-1102, Beckman).

### Statistical analysis

The Wilcoxon test and t-test were applied to compare data between two groups. The R software packages “survminer” and Kaplan-Meier survival plot were used to estimate overall survival rates between two groups. The survival analysis Cox regression was done using the R software package “survival”. Heatmaps were generated using the R package “pheatmap”. Data visualization was mainly performed using the ggplot2 R software (v4.1.2). Statistical analysis of the data was conducted using Graphpad Prism 8.0. Data were presented as mean ± standard deviation. Normality and homogeneity of variance were assessed before conducting the analysis. One-way ANOVA with Tukey’s post hoc test was applied for comparisons among multiple groups. *P*-value < 0.05 indicated statistical significance.

## Results

### Cluster analysis of immunoinfiltrating subtypes of gastric cancer

**Fig. 1:**
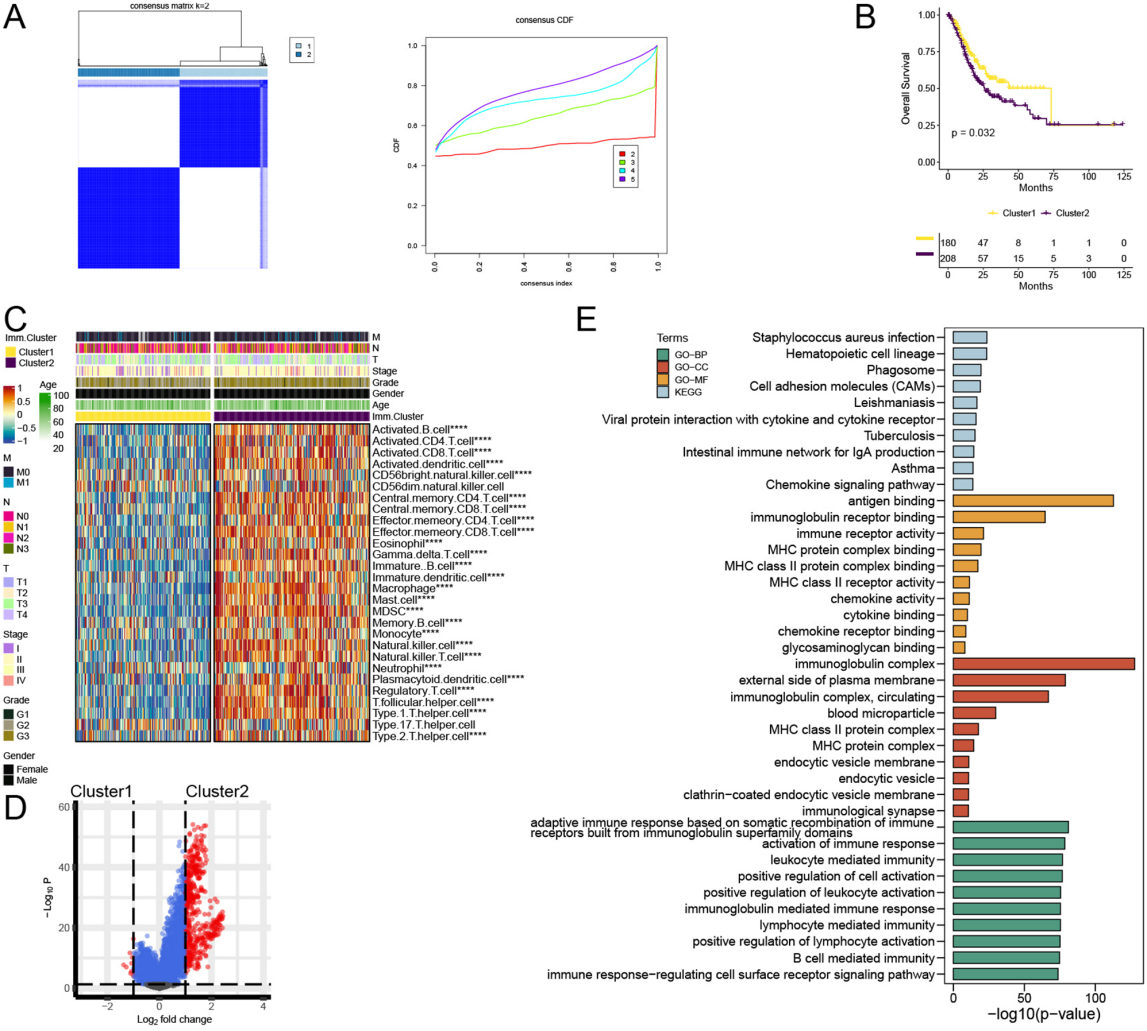
Gastric cancer subtypes were constructed by unsupervised cluster analysis based on the level of immune infiltration. (**A**) The gastric cancer subtypes associated with immune invasion were identified by unsupervised cluster analysis (cluster1 and cluster2). (**B**) Survival analysis plot cluster. (**C**) Immune cell infiltration cluster. (**D**) Volcano cluster. (**E**) Functional enrichment analysis. ****P < 0.0001

Based on the 28 immune cells infiltration in ssGSEA, we used the ConsensusClusterPlus R package to obtain the immunoinfiltration-associated cluster1 and cluster2 subtypes of gastric cancer (Fig. 1A). Overall survival was shorter in patients with cluster2 gastric cancer subtype compared with cluster1 subtype (Fig. 1B). Immune infiltration level of patients with cluster2 gastric cancer subtype was higher than that in the cluster1 subtype (Fig. 1C). Analysis of differences between groups revealed significant changes in 500 genes between the cluster1 and cluster2 subtypes of gastric cancer (Fig. 1D). Differential genes were used for functional prediction. GO showed significant enrichment of staphylococcus aureus infection, antigen binding and immunoglobulin complex (Fig. 1E). Adaptive immune response based on somatic recombination of immune receptors built from immunoglobulin superfamily domains were significant enriched in KEGG (Fig. 1E). These results indicate that cluster2 patients with gastric cancer subtype were related to the function of differential immune genes.

### CCDC80 is one of the biomarkers of poor prognosis in gastric cancer

**Fig. 2:**
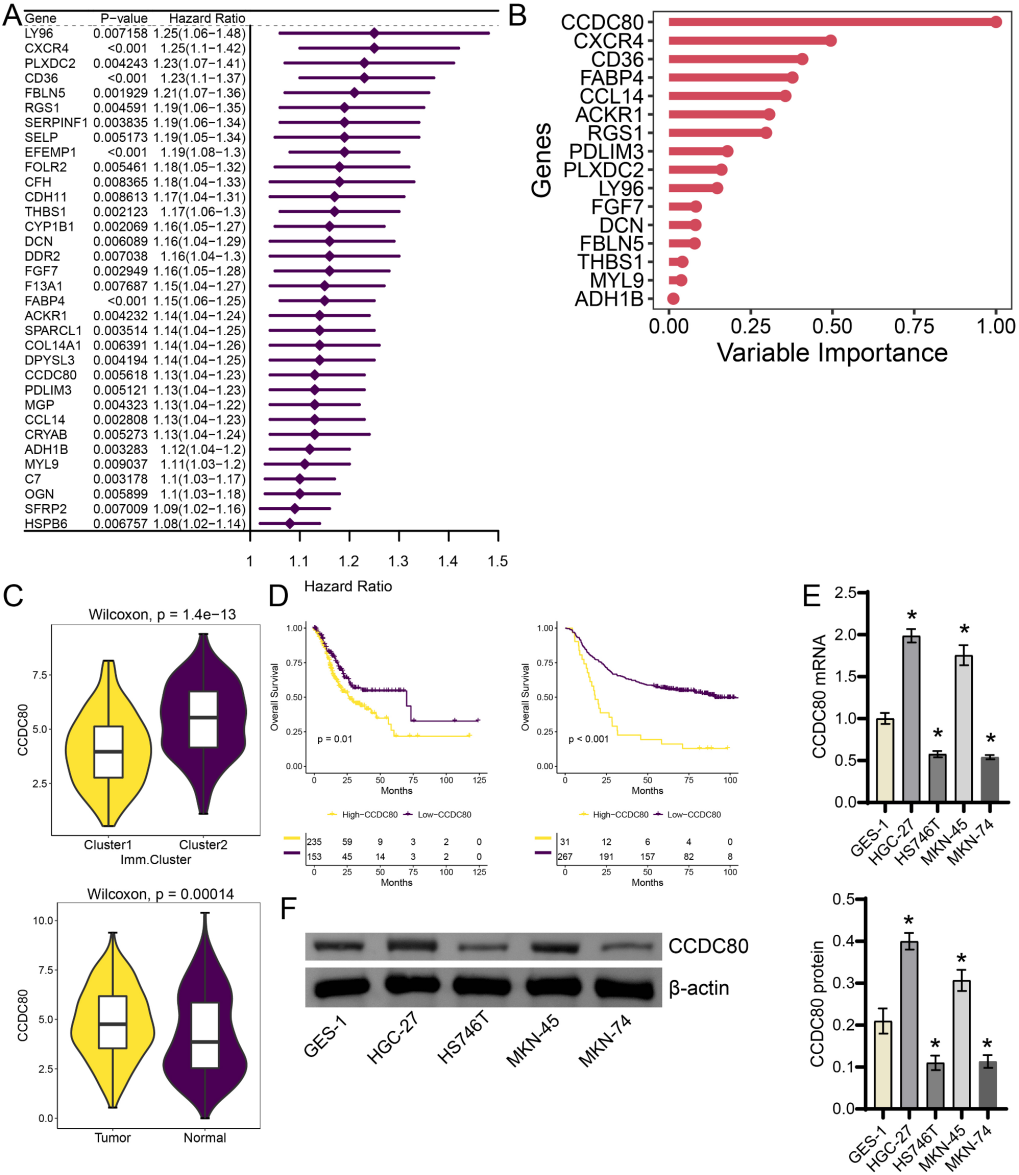
CCDC80 predicted poor prognosis in gastric cancer. (**A**) Single factor analysis cluster. (**B**) Random forest analysis using 34 prognostic-related genes. (**C**) Boxplot of CCDC80 cluster. (**D**) Survival plot of CCDC80. (**E-F**) RT-qPCR and western blot were used to detect the CCDC80 expression in GES-1, HGC-27, HS746T, MKN-45 and MKN-74 cells. *P < 0.05 vs. GES-1

Single factor analysis based on TCGA data set screened 34 differential genes (Fig. 2A). Based on random forest analysis using 34 prognostic-related genes, the number of genes was reduced to 16 (Fig. 2B). Among these 16 genes, CCDC80 has the highest priority (Fig. 2B). Compared with cluster1, CCDC80 was significantly higher expressed in cluster2 subtype (Fig. 2C). Compared with Normal group, CCDC80 was significantly overexpressed in patients with gastric cancer (Fig. 2C). Survival analysis in view of the TCGA dataset and GSE62254 showed that patients with high-expression of CCDC80 had a lower survival rate (Fig. 2D). Therefore, CCDC80 was selected for further analysis. Compared with GES-1 cells, mRNA, and protein levels of CCDC80 were higher in HGC-27 and MKN-45 cells, and significantly lower in HS746T and MKN-74 cells (Fig. 2E-F, Supplement Fig. 1). Therefore, CCDC80 was the potential biomarker for poor prognosis in gastric cancer.

### Silencing CCDC80 inhibited malignant characterization of HGC-27 and MKN-45 cells

**Fig. 3:**
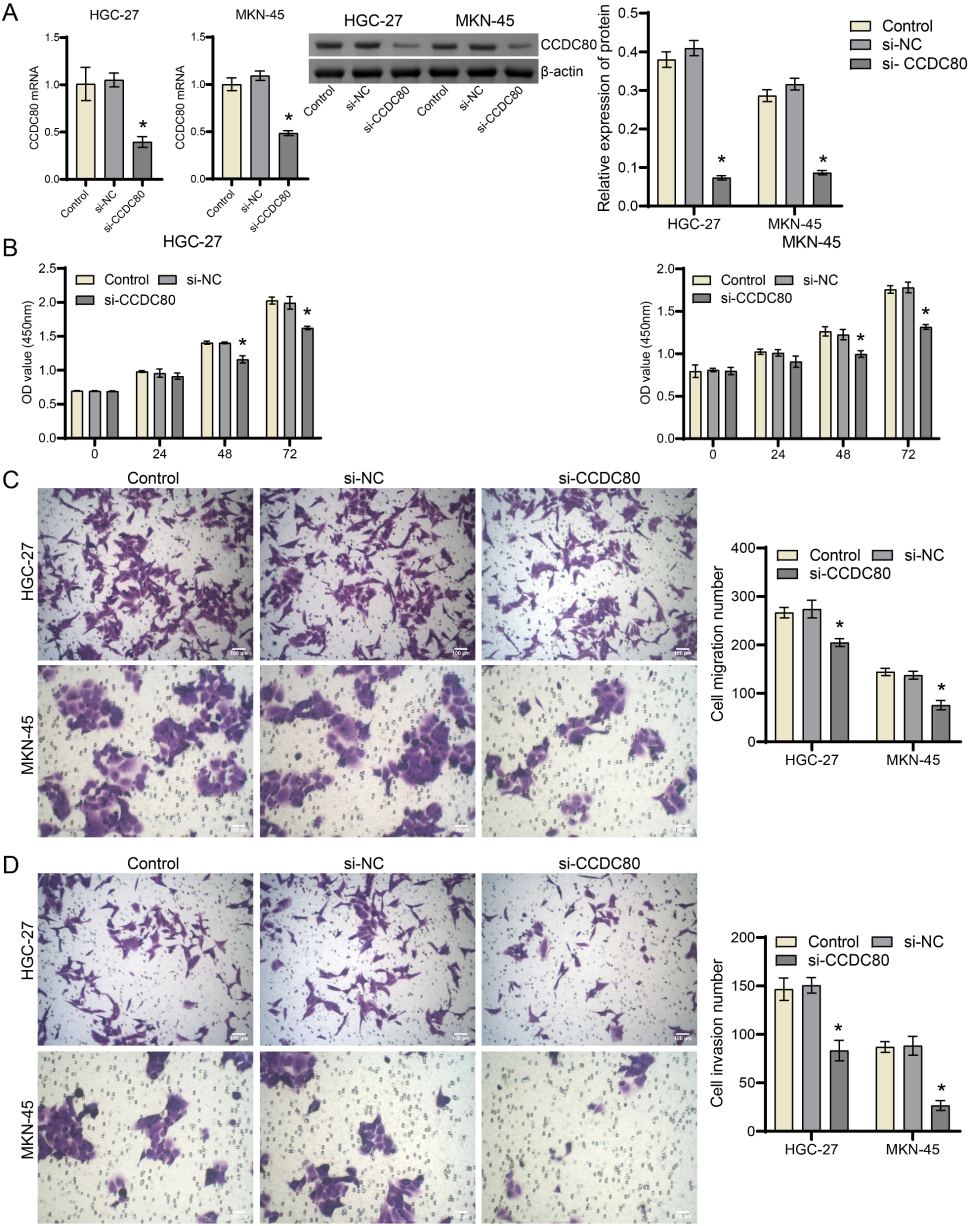
Silencing CCDC80 inhibited the malignant characterization of HGC-27 and MKN-45. (**A**) RT-qPCR and western blot testing for the expression of CCDC80. (**B**) CCK-8. (**C - D**). Transwell. *P < 0.05 vs. si-NC

Subsequently, we transfected HGC-27 and MKN-74 cells with si-CCDC80 to investigate its role in the characterization of gastric cancer. CCDC80 mRNA and protein levels were downregulated in the si-CCDC80 than si-NC groups, confirming the successful construction of CCDC80-silenced HGC-27 and MKN-74 cells (Fig. 3A, Supplement Fig. 1). In addition, the silencing of CCDC80 significantly inhibits the proliferation, migration, and invasive ability of HGC-27 and MKN-74 cells (Fig. 3B-D). The above results demonstrate that the silence of CCDC80 inhibited the malignant characteristics of gastric cancer cells.

### Silencing CCDC80 inhibits tumorigenesis of gastric cancer cells

**Fig. 4:**
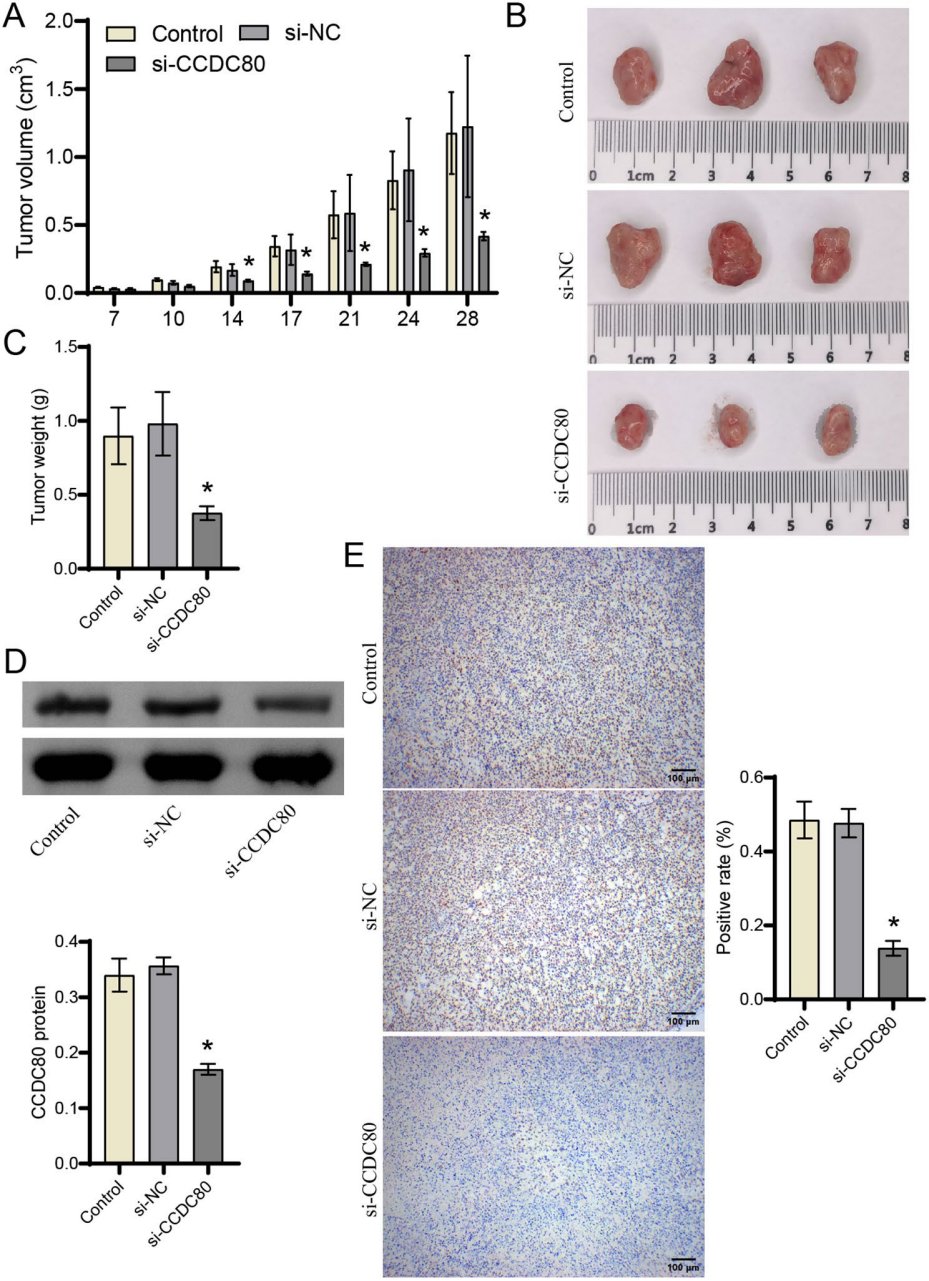
Silencing CCDC80 inhibits the development of gastric cancer. (**A**) Tumor volume. (**B**) Tumor observation. (**C**) Tumor weight. (**D**) CCDC80 expression was detected by western blot. (**E**) The expression of PCNA was detected by IHC. *P < 0.05 vs. si-NC

Next, we further investigated the role of CCDC80 silence in the development of gastric cancer cells in nude mice. As the subcutaneous tumor time increased, the tumor volume gradually increased (Fig. 4A). From day 14 to day 28, the tumor volume was significantly reduced in si-CCDC80 than si-NC groups (Fig. 4A). The observation of the tumor on day 28 was consistent with the changes in volume (Fig. 4B). At the same time, the tumor weight in si-CCDC80 group was lower than si-NC group, consistent with the changes in volume (Fig. 4C). The examination of tumor tissue showed that CCDC80 and PCNA proteins expression in si-CCDC80 group was lower than si-NC group (Fig. 4D-E, Supplement Fig. 1). Silencing CCDC80 inhibited the development and proliferation of subcutaneous gastric cancer tumors.

### CCDC80 is associated with immune invasion of gastric cancer

**Fig. 5:**
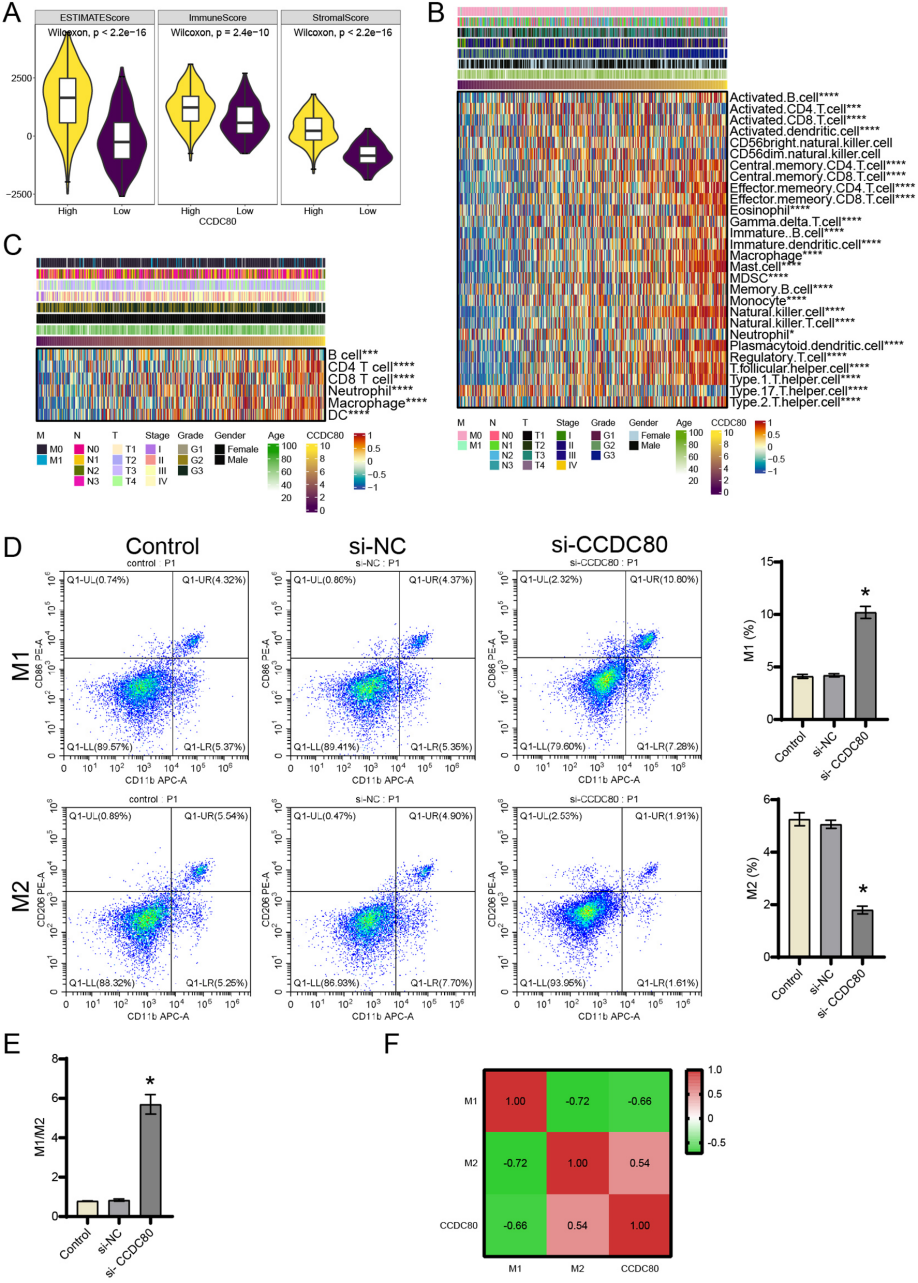
Correlation analysis of CCDC80 and immunoinfiltration in gastric cancer. (**A**) Tumor microenvironment immune score. (**B**) Heatmap of immuneinfiltration in ssGSEA. (**C**) Heatmap of immuneinfiltration in TIMER. ***P < 0.001, ****P < 0.0001. (**D**) The ratio of M1 and M2 in tumor tissues was analyzed by flow cytometry. (**E**) Value of M1/M2. (**F**) Correlation in the level of M1, M2 and CCDC80 was analyzed by spearman

Relationship between CCDC80 expression and immune infiltration was explored in gastric cancer. The tumor microenvironment immune scoring showed a significant increase in ESTIMATE, immune, and stromal scores in the group with high CCDC80 expression (Fig. 5A). Immunoinfiltration analysis based on ssGSEA showed that high CCDC80 expression was related to the most immune cell’s infiltration (Fig. 5B). Immunoinfiltration analysis based on TIMER further confirmed that immune infiltration of CD4 + T cells, B cells, neutrophils, CD8 + T cells, dendritic cells, and macrophages was seriously in patients with high CCDC80 expression (Fig. 5C). The expression levels of immune checkpoint-related molecules, including HLA − DQA1, HLA − DQB1, HLA − DQA2, HLA − DQB2, HLA − DRA, HLA − DRB1, HLA − DPB1, HLA − DPA1, ITGB2, ICAM1, SELP, PDCD1LG2, SLAMF7, BTN3A2, BTN3A1, CD276, CD80, CD28, TNFSF4, IL1B, CXCL9, CCL5, VEGFB, CX3CL1, TGFB1, VEGFA, CD70, CD40LG, IL10, IL12A, IL13, GZMA, PRF1, HMGB1, ENTPD1, TIGIT, PDCD1, IL2RA, TNFRSF4, CD27, TNFRSF9, ICOS, BTLA, KIR2DL3, KIR2DL1, EDNRB, CD40, ADORA2A, TLR4, and HAVCR2 (*P* < 0.0001), were significantly changes with CCDC80 expression (Supplement Fig. 2). We utilized flow cytometry to analyze the polarization of macrophages in tumor tissues and found that silencing CCDC80 promoted M1 polarization while inhibited M2 polarization (Fig. 5D). Silencing CCDC80 led to an increase in the M1/M2 ratio (Fig. 5E). Correlation analysis revealed a positive correlation between CCDC80 and M2 polarization, and a negative correlation with M1 polarization (Fig. 5F). These results demonstrate that high CCDC80 expression was positively related to tumor microenvironment immune infiltration in gastric cancer patients.

### CCDC80 was associated with somatic mutations in patients with gastric cancer

**Fig. 6:**
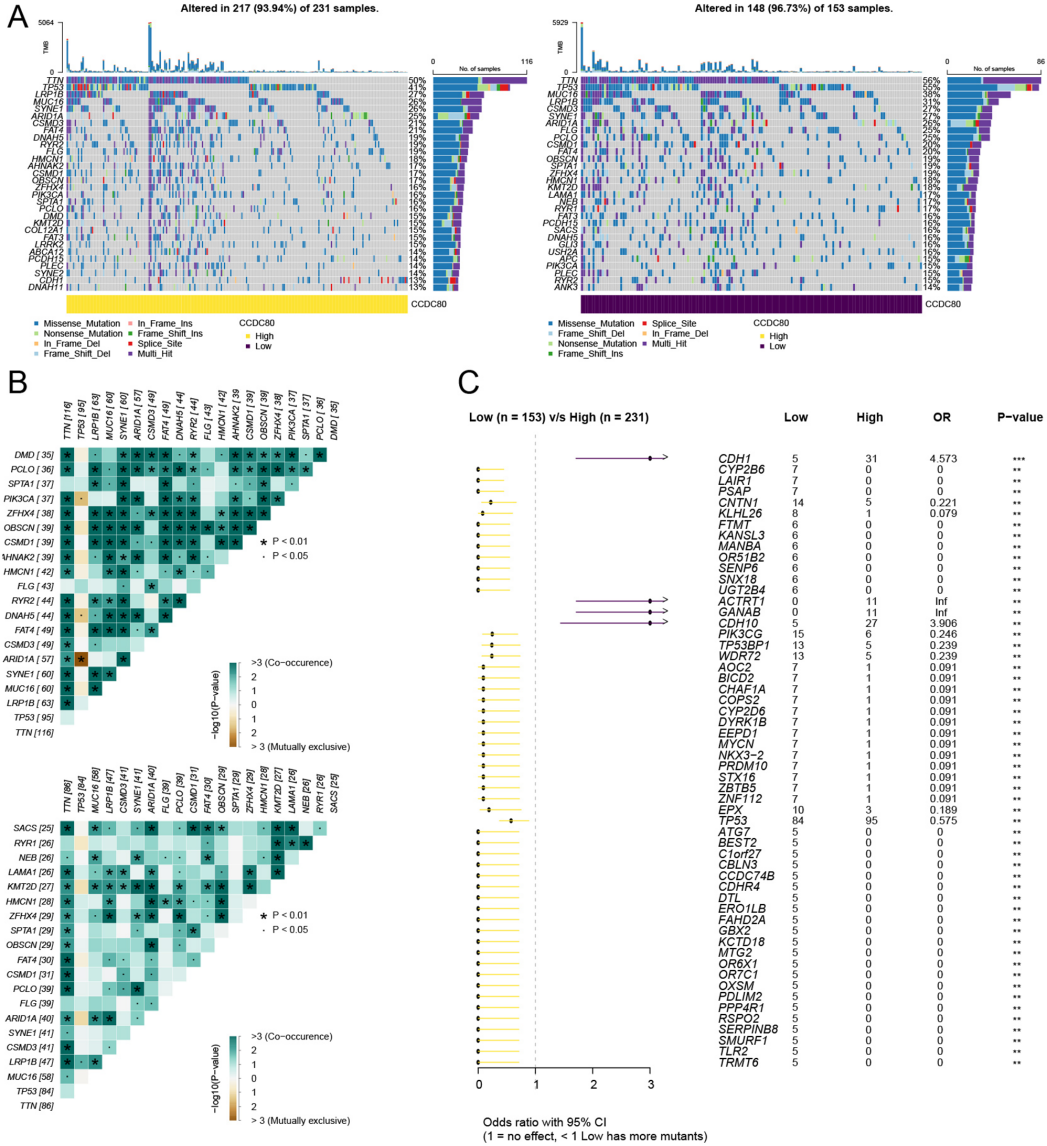
Analysis of CCDC80 expression and gene mutation in patients with gastric cancer. (**A**) Oncoplot of Top30 high and Top30 low genes. (**B**) The somaticInteractions function analyzed the mutually exclusive or coexisting relationships between mutated genes. (**C**) Prediction of mutation probability of top 30 genes between high and low CCDC80 groups. *P < 0.05

Subsequently, we further analyzed the relationship between CCDC80 and somatic gene mutations in gastric cancer patients. Among the 231 samples in the High-CCDC80 group, 93.94% showed gene mutations, while in the Low-CCDC80 group, 96.73% of the 153 samples showed gene mutations (Fig. 6A). The heatmap further revealed the co-occurrence or exclusivity of the mutated genes (Fig. 6B). Additionally, compared to the Low-CCDC80 group, the genes CDH1, ACTRT1, GANAB, and CDH10 were found to have a higher likelihood of mutation in the High-CCDC80 group (Fig. 6C). These findings demonstrate that high CCDC80 expression had mutations with CDH1, ACTRT1, GANAB, and CDH10 genes in gastric cancer, which might be related to the malignancy of gastric cancer with high CCDC80 expression.

### Function prediction of CCDC80 in patients with gastric cancer

**Fig. 7:**
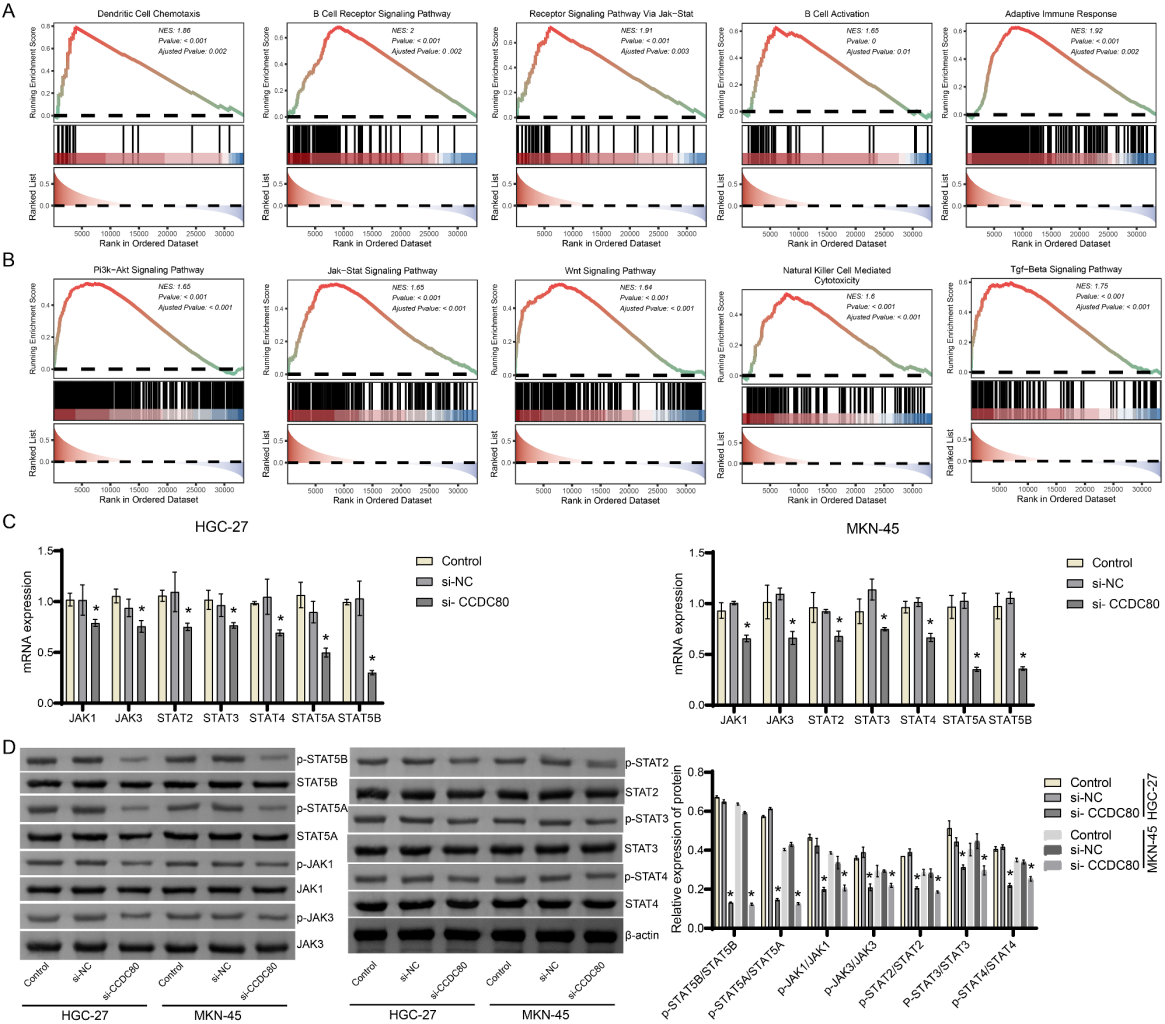
Function analysis of CCDC80 in patients with gastric cancer. **A-B**. The function of CCDC80 in patients with gastric cancer was analyzed in GO and KEGG database. **C-D**. The expression of JAK1, JAK3, STAT2, STAT3, STAT4, STAT5A, and STAT5B in HGC-27 and MKN-45 cells was detected by RT-qPCR and western blot. *P < 0.05 vs. si-NC.

Function prediction of CCDC80 in GO database proved that dendritic cell chemotaxis, B cell receptor signaling pathway, JAK-STAT cascade, B cell activation and adaptive immune response were enriched (Fig. 7A). The analysis in KEGG database proved that PI3K-Akt signaling pathway, JAK-STAT signaling pathway, Wnt signaling pathway, Natural killer cell mediated cytotoxicity and TGF-beta signaling pathway were enriched (Fig. 7B). Further analysis of cell validation reveals that silencing CCDC80 inhibited the expression of JAK1, JAK3, STAT2, STAT3, STAT4, STAT5A, and STAT5B genes and proteins in HGC-27 and MKN-45 cells (Fig. 7C and D, Supplement Fig. 1). These findings demonstrate that CCDC80 might regulated JAK-STAT signaling pathway in gastric cancer.

## Discussion

Our research is based on the bioinformatics analysis of the TCGA dataset, which shows that CCDC80 was significantly overexpressed in the group of gastric cancer and was related to tumor immune infiltration level. In ApcMin/+ mice and chemically-induced colorectal cancer models, Dro1/Ccdc80 has been proven to be an effective suppressor of colorectal cancer [29, 30]. In addition, cl2/ccdc80(-/-) mice are prone to thyroid adenomas and ovarian cancer [31], may be a tumor suppressor gene in thyroid cancer [32]. On the other hand, CCDC80 can be used to predict cancer stem cell-related features in colorectal adenocarcinoma and ovarian cancer prognosis [11, 16]. CCDC80 is characterized by promoter hypomethylation and upregulation in esophageal cancer [33]. The above results indicate that CCDC80 functions as an oncogene or a tumor suppressor gene in cancer, potentially distinguishing it among different types of cancer. Cell experiments with gastric cancer cells confirmed high expression of CCDC80 in gastric cancer, proving it might serve as a biomarker for gastric cancer patients.

CCDC80 mediates focal adhesion kinase (FAK) regulation of B16F10 melanoma cell migration [34]. The overexpression of CCDC80 inhibits the growth of butylated hydroxyanisole-induced CRC cells through suppress the ERK1/2 activation [35]. The deletion of CCDC80 can hinder the growth-inhibitory effect of LATS1/2 (Hippo pathway kinases) deficiency in MC38 cells [36]. The above studies have confirmed that CCDC80 is involved in the malignant characteristics of tumors. Our results show that silencing CCDC80 inhibits the malignant characteristics (HGC-27, MKN-45). The results also preliminarily confirmed the inhibitory effect of si-CCDC80 on tumor formation (3 nude mice per group). Although the current number of animals meets the requirements for testing and statistics, the limitation of this study is that each group of nude mice only has 3 individuals. It is recommended to further increase the number of animals [37] or perform the knockout mouse model [38] to explore the role of CCDC80 in gastric cancer in depth. Therefore, CCDC80, as an oncogene, played a role in gastric cancer cells and might be a potential cure target.

By CIBERSORT, the proportion of tumor infiltrating memory B cells, follicular helper T cells and the activated NK cells in high-CCDC80 group was higher than that in low-CCDC80 group [39]. Atopic dermatitis whole genome association study and downstream analysis suggest that the expression signals of CCDC80 overlap significantly in enhancers of skin cells and immune cells, particularly CD4 T cells [40]. Patients with CCDC80-positive ovarian cancer have higher CD4 + memory-resting cells infiltration [41]. Our results found that GC patients with high-expression of CCDC80 subtype showed an unfavorable prognosis, while the ssGSEA analysis revealed that higher levels of immune infiltration in high-expression of CCDC80 subtype. This result may be consistent with the above studies. This phenomenon suggests that CCDC80 may play a complex role in T cell immunity in gastric cancer, but the specific mechanisms of action still need further exploration.

In addition, vactosertib combined with nal-IRI/5-FU/LV improved survival by inhibiting CCDC80 invasion in pancreatic cancer [42]. The exosome delivery system derived from parental cells aims to simultaneously deliver CCDC80-targeting siRNA and increase the chemotherapy sensitivity of distant colon cancer liver metastasis mouse models and xenograft mouse models originated from patients [43]. The above studies confirmed that CCDC80 was related to immune infiltration in cancer patients and was involved in cancer chemotherapy. Our results found that high-expression of CCDC80 was positive correlation with immune invasion of B cell, T cell CD4, T cell CD8, Neutrophil, Macrophage and DC, as well as the immune function and JAK-STAT signaling pathways. Silencing CCDC80 also inhibited the M2 polarization and JAK-STAT pathway in cells and tumor tissue of gastric cancer. Therefore, CCDC80 is highly expressed as the oncogene in highly immunoinvasive gastric cancer subtypes. CCDC80 may be a potential target to promote chemotherapy sensitivity in gastric cancer patients with tumor microenvironment immune restriction.

### Electronic supplementary material

Below is the link to the electronic supplementary material.

Supplementary Material 1

Supplementary Material 2

Supplementary Material 3

## Data Availability

All data found in this study are included in the manuscript or are available upon request by contact with the first author or corresponding author. Raw data of GSE62254 and TCGA can be download in Supplementary Dataset File.
