# Supplementary material for: Silencing immune-infiltrating biomarker CCDC80 inhibits malignant characterization and tumor formation in gastric cancer
Source: BMC Cancer. 2024 Jun 13;24:724. doi: 10.1186/s12885-024-12451-y (PMC11170897; doi:10.1186/s12885-024-12451-y)
Supplement: Supplementary file 3 — Supplementary Material 3 [file 12885_2024_12451_MOESM3_ESM.docx]

**Supplementary materials**


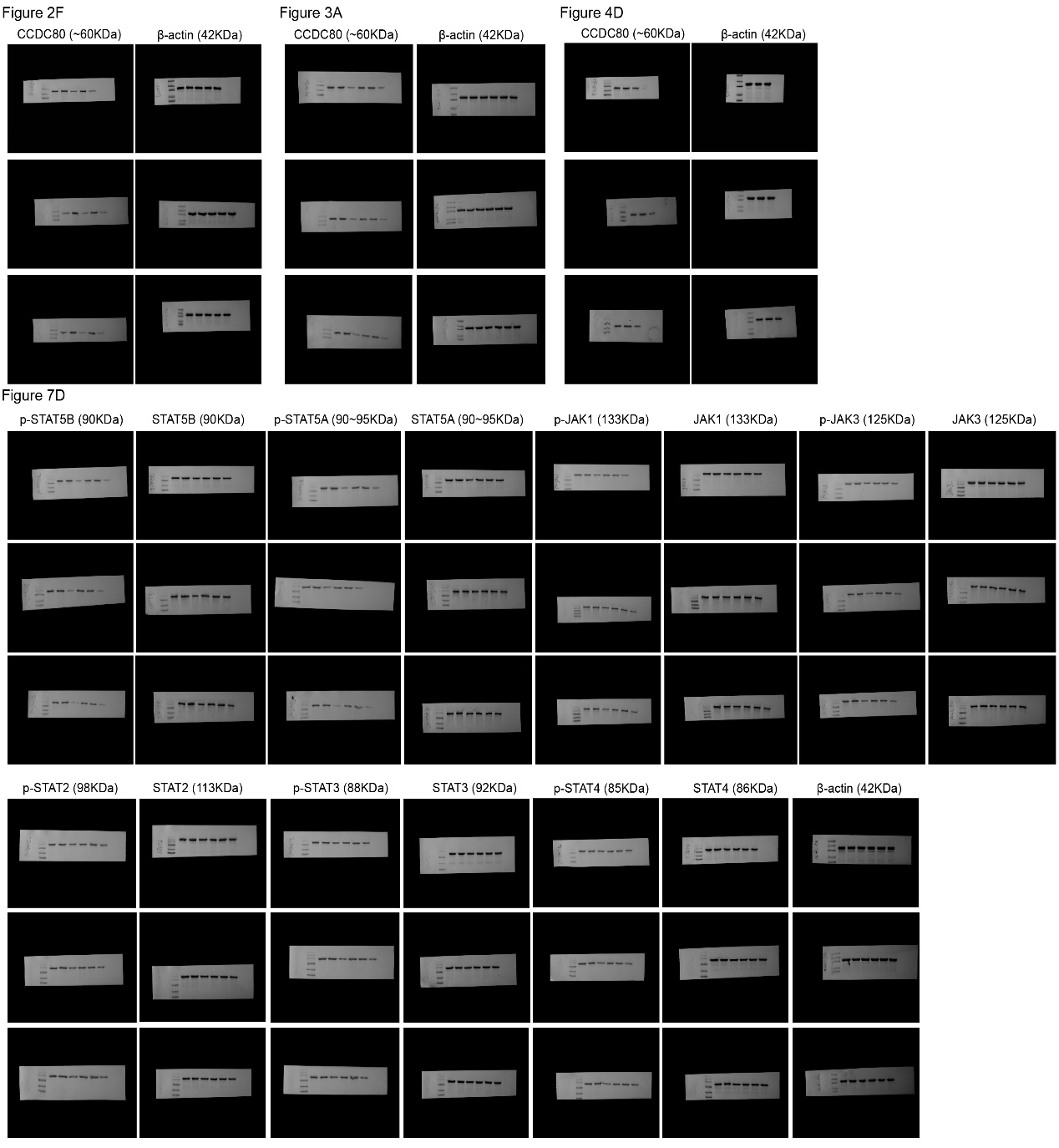


**Supplement Figure 1 Western blot of the original blots.**


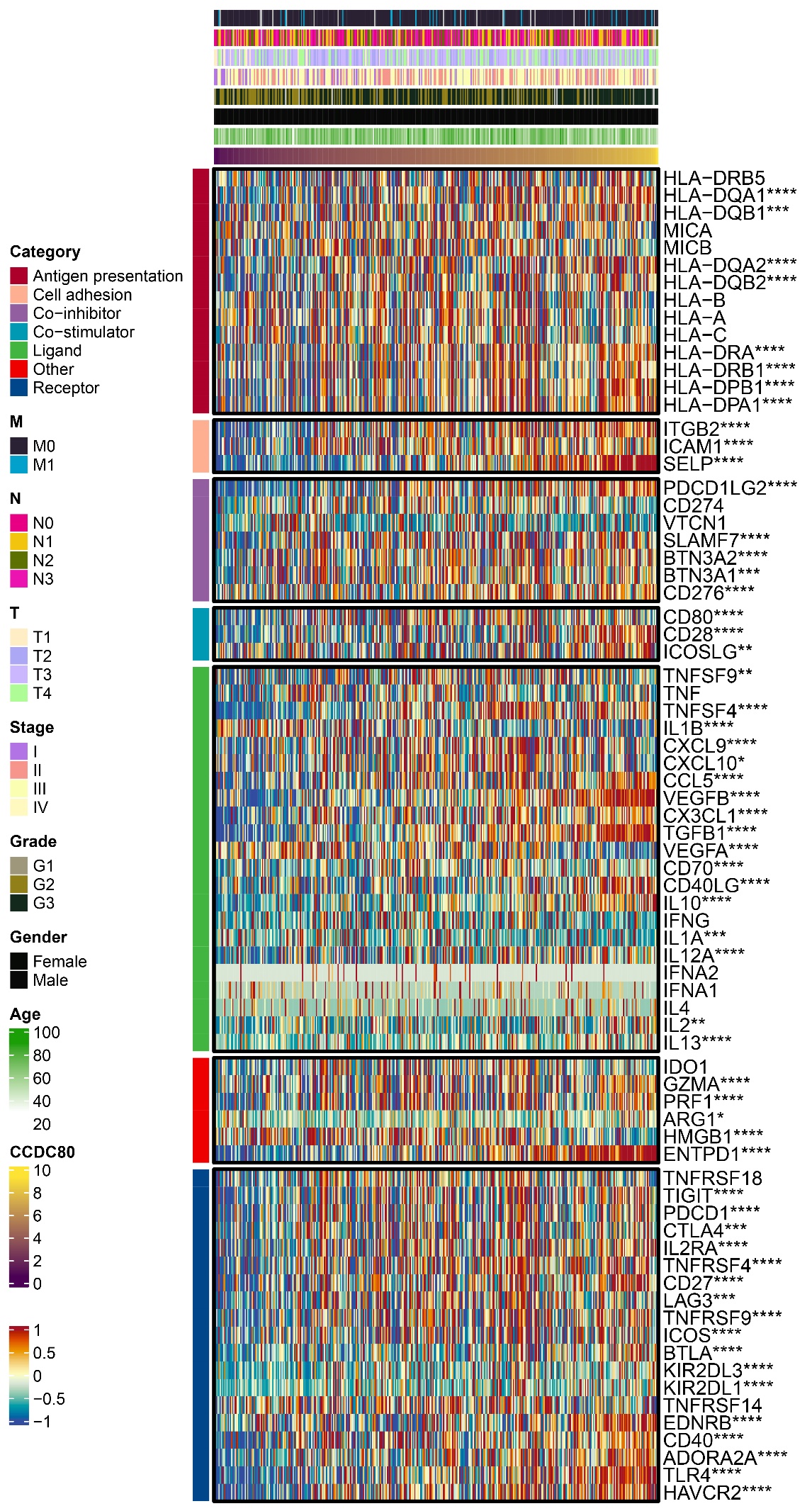


**Supplementary Figure 2 The expression levels of immune checkpoint-related molecules in different groups.** *P<0.05, **P<0.01, ***P<0.001, ****P<0.0001.
